# Optimal numbers of residues in linkers of DNA polymerase I, T7 primase and DNA polymerase IV

**DOI:** 10.1038/srep29125

**Published:** 2016-07-01

**Authors:** Yi-Ben Fu, Zhan-Feng Wang, Peng-Ye Wang, Ping Xie

**Affiliations:** 1 Key Laboratory of Soft Matter Physics and Beijing National Laboratory for Condensed Matter Physics, Institute of Physics, Chinese Academy of Sciences, Beijing 100190, China

## Abstract

DNA polymerase I (PolI), T7 primase and DNA polymerase IV (Dpo4) have a common feature in their structures that the two main domains are connected by an unstructured polypeptide linker. To perform their specific enzymatic activities, the enzymes are required to rearrange the position and orientation of one domain relative to the other into an active mode. Here, we show that the three enzymes share the same mechanism of the transition from the inert to active modes and use the minimum numbers of residues in their linkers to achieve the most efficient transitions. The transition time to the finally active mode is sensitively dependent on the stretched length of the linker in the finally active mode while is insensitive to the position and orientation in the initially inert state. Moreover, we find that for any enzyme whose two domains are connected by an unstructured flexible linker, the stretched length (*L*) of the linker in the finally active mode and the optimal number (*N*_*opt*_) of the residues in the linker satisfy relation *L* ≈ *αN*_*opt*_, with *α* = 0.24–0.27 nm being a constant insensitive to the system.

A lot of proteins or enzymes have the structures with two domains being connected by an unstructured polypeptide linker. To perform the specific enzymatic activity, the enzyme is required to rearrange the position and/or orientation of one domain relative to the other domain into an active mode by stretching the flexible linker. Thus, it is an interesting topic of how the linker affects this rearrangement. Here, we take DNA polymerase I (PolI), bacteriophage T7 primase and DNA polymerase IV (Dpo4) as examples to study the effect of the linker that connects the two domains of the enzyme on this transition from the inert to active modes, determining how many residues in the linker are required to make the enzyme achieve the most efficient transition.

PolI is a well-characterized enzyme involved in DNA replication and repair. During the replication of the lagging strand DNA, it plays a particularly important role in the processing of Okazaki fragment^1^,^2^. PolI is composed of a single polypeptide chain consisting of about 930 residues. It possesses two main domains—a core domain, including a polymerase domain and 3′-5′ exonuclease domain (3′-5′ domain), and 5′-nuclease domain (5′-domain) (Fig. 1a)^3^,^4^,^5^. There is also a polypeptide linker of 16 residues that tethers the 3′-5′ domain and 5′-domain. The polymerase domain, pictured as a right hand, is composed of thumb, fingers and palm^6^,^7^,^8^. PolI catalyzes DNA synthesis initially from a primer along the template strand. The 3′-5′ domain is able to proofread those unmatched deoxyribonucleotides^9^. In the replication of the lagging strand, the downstream primer gets closer and closer to the polymerase activity site, and eventually the downstream primer detaches from the DNA template to generate a flap DNA. The 5′-nuclease, which has a high affinity to the flap DNA^10^,^11^, can catch the flap DNA and cut down the primer, leaving a nick for DNA ligase to seal^12^.

T7 primase, which can synthesize RNA primers, is located in the N-terminal half of the multifunctional gene 4 helicase-primase protein^13^. The primase domain is composed of an RNA polymerase domain (RPD) and a zinc-binding domain (ZBD). The two domains are connected by an unstructured polypeptide linker of 16 residues^14^. ZBD is essential for the recognition of a special sequence, 5′-GTC-3′^15^, where T7 primase uses the diribonucleotide pppAC to catalyze the synthesis of the functional tetraribonucleotides pppACCC, pppACCA, pppACAC^16^,^17^.

Dpo4 is a prototypical Y-family DNA polymerase that can catalyze translesion DNA synthesis^18^. It is composed of a core domain and a little finger (LF)^19^. The core domain possesses thumb, fingers and palm domains^20^. A polypeptide linker of 11 residues connects the thumb and LF domains. When Dpo4 binds DNA into Dpo4-DNA binary complex, Dpo4 undergoes a dramatic conformational change, with the LF domain moving and rotating relative to the core domain and in addition making a 131° azimuthal rotation^21^.

To study the effect of the linker on the dynamics of the transition from the inert to active modes in these enzymes, we first determine the elasticity of the linker by using atomic molecular dynamic (MD) simulations. Then, with the determined elasticity of the linker we study the dynamics of the transition by using the simplified model of the system. Our results show that the wild-type (wt) linkers of the three enzymes contain the minimum numbers of residues to achieve the most efficient transitions. More interestingly, we find that for any enzyme whose two domains are connected by an unstructured flexible linker, the stretched length (*L*) of the linker in the finally active state and the optimal number (*N*_*opt*_) of the residues in the linker satisfy relation *L* ≈ *αN*_*opt*_, with *α* = 0.24–0.27 nm being a constant insensitive to the enzyme.

## Results and Discussion

### Force-extension relation of the linker of PolI

**Figure 1:**
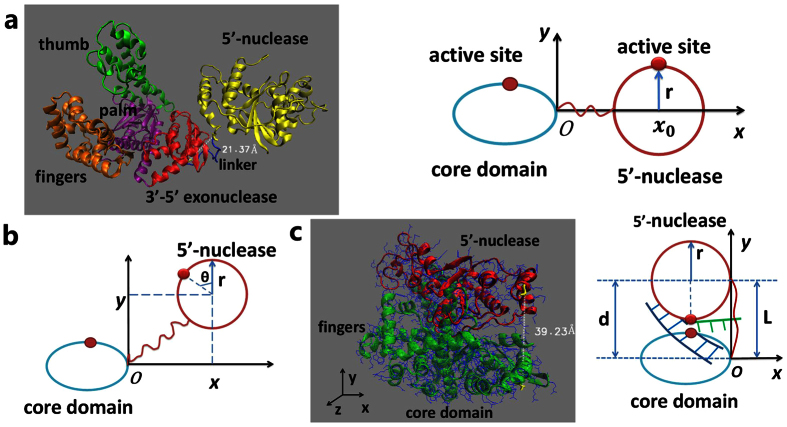
Structures and schematic diagrams of PolI in initial, intermediate and final states. (**a**) Left panel shows the crystal structure of PolI (1TAQ.pdb). Right panel is schematic diagram of PolI in the initial condition. (**b**) Schematic diagram of PolI in the intermediate state, with its center-of-mass position and rotation angle being described by (*x*, *y*, *θ*). (**c**) Left panel shows the most probable position and orientation of the 5′-domain relative to the core domain in the finally active mode. The 5′-domain is in such position and orientation that the 5′-nuclease active site is located as close as possible to the polymerase active site of the core domain. Moreover, the residues in the 5′-domain are not allowed to overlap with those of the core domain due to the spatial exclusion. In the structure shown here, the DNA substrate can be justly located in the space formed by the 5′-domain and core domains. The end-to-end distance of the linker is estimated to be 39.23 Å and the 5′-domain is rotated by about 180°. Right panel is the schematic diagram of the structure shown in the left panel.

During DNA replication by PolI, as the polymerase active site of the core domain approaches the 5′-flap DNA substrate, the 5′-domain would transit from the position relative to the core domain that is distant from the cleavage site on the 5′-flap DNA substrate to the position where the 5′-nuclease site becomes coincident with the site of the scissile phosphate diester bond of the flap (Fig. 1)^22^. During this transition, the linker is stretched. Thus, to study the transition dynamics, we should determine the elasticity of the linker by using all-atom MD simulations (see Methods). We use two ways to simulate the extension of the linker under the external force acting on the two ends of the linker. One way is to simulate the whole molecule of PolI and the other way is to delete all domains of PolI and keep only the linker, as usually done in the literature^23^. The latter way implicitly assumes that there is no interaction between the two domains that the linker connects. By comparing the force-extension relations obtained with the two ways of simulations, we can determine if there is the interaction between the two domains.

**Figure 2:**
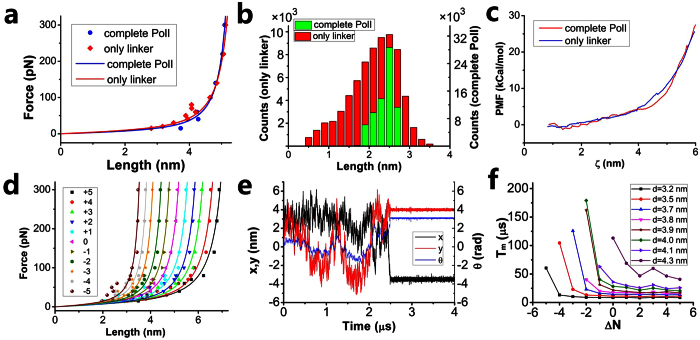
Results for PolI. (**a**) Force-extension relations of the linker simulated with complete PolI and with only linker (dots). Lines are fit curves with WLC model. (**b**) Distributions of the end-to-end distance of the linker simulated with complete PolI and with only linker. (**c**) PMF versus the end-to-end distance of the linker (*ζ*) simulated with complete PolI and with only linker. (**d**) Force-extension relations of 11 linkers. (**e**) A typical trajectory of the motion and rotation of the 5′-domain relative to the core domain. (**f**) Mean transition time *T*_*m*_ versus Δ*N* for different values of *d*.

To make simulations of the complete PolI (1TAQ.pdb)^3^, we adjust the direction of the molecule to make the line connecting the centers of the core and 5′-domains along the *x* axis. We fix some residues (residues 278–289) of the 5′-domain and impose a series of constant forces on some residues (residues 306–448) of the core domain along the *x* axis. We then calculate the distance between the two terminal alpha carbons of the linker (residues 290 and 305) after the system reaches equilibrium. To make simulations of only the linker, we keep residues 289 through residue 306, where the residues 290–305 constitute the linker. We adjust the line connecting residues 289 and residue 306 along the *x* axis. We fix residue 289 and impose a series of constant forces on residue 306 along the *x* axis. We then calculate the distance between the two terminal alpha carbons of the linker after the equilibrium. Each simulation is carried out in a time period of 60 ns. The calculated data of the force-extension relations of these linkers can be fitted by the worm-like-chain (WLC) model


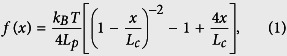


where *L*_*c*_ is the contour length and *L*_*p*_ is the persistence length of the linker (Fig. 2a). The results show that the force-extension curve of the linker with the complete PolI nearly overlaps that with only the linker, implying that nearly no interaction exists between the two domains.

To further test that no interaction exists between the two domains, we use two other methods. One method is to make statistical studies of the end-to-end distance distribution of the linker without the external force by using MD simulations of the complete PolI and of only the linker. The results show that although the end-to-end distance distribution of the single linker is wider than that of the linker in PolI, the peaks of the two distributions occur at the same distance (Fig. 2b), implying that neither attraction nor repulsion exists between the two domains. The fact that distribution for the single linker is wider than that for the linker in PolI is due to the smaller damping in the former case than in the latter case. The other method is to calculate the potential of mean force (PMF) during the pulling of the linker with the complete PolI and with only the linker by using the umbrella sampling method (see Methods). The spring constant is taken to be 1000 kJ ⋅ mol^−1^ ⋅ nm^−2^ and pulling rate is 0.001 nm ⋅ ps^−1^. We have checked that provided the pulling rate is smaller than 0.01 nm/ps, the change in the pulling rate has no effect on PMF (Fig. S1). We have also checked that the change in the pulling direction has no effect on PMF (Fig. S1). The results show that the two curves of PMF versus the end-to-end distance of the linker are also nearly coincident (Fig. 2c). Thus, in our calculations, we can make simulations of only the linker to obtain the force-extension relation of the linker of PolI.

Here, we calculate the force-extension curves for 11 linkers of different numbers of residues, which are denoted by Δ*N* = −5, …, +5. Δ*N* = 0 corresponds to the wild-type (wt) linker of 16 residues (deviation from 16 by 0). Δ*N* = −1, …, −5 correspond to the linkers with 1, …, 5 residues deleted from the terminus (residue 305) of the wt linker (deviations from 16 by −1, …, −5), respectively. Δ*N* = + 1, …, +5 correspond to the linkers with 1, …, 5 residues added to the terminus (residue 305) of the wt linker (deviations from 16 by 1, …, 5), respectively, where the types of the added residues are chosen randomly. The calculated data of the force-extension relations of these linkers can also be fitted by the worm-like-chain (WLC) model, i.e., Eq. (1) (Fig. 2d).

### Dynamics of transition of 5′-domain from inert to active modes in PolI

As done before^22^, we approximate the core domain of PolI as an ellipsoid and the 5′-domain as a sphere of radius *r* (right panel of Fig. 1a). We consider the motion of the 5′-domain relative to the core domain to study the relative motion between the two domains. Here, we confine to two dimensions *x* and *y* in the plane of the paper surface, for the motion in the *z* direction that is perpendicular to the *xoy*-plane has no effect on our conclusion of this work (see below). The coordinate *xoy* is shown in Fig. 1a (right panel) and 1b. Thus, the motion of the 5′-domain is characterized by its center-of-mass position (*x*, *y*) and the rotation by its rotational angle *θ*, where *θ* is defined as the rotation angle of the line connecting the center-of-mass position and the active site relative to the *y* axis, with *θ* = 0 in the initial state. According to the structural data (1TAQ.pdb), the initial state of the 5′-domain is at (*x*, *y*, *θ*) = (*x*_0_, 0, 0) (*x*_0_ > *r*) (Fig. 1a, right panel). At an intermediate state (*x*, *y*, *θ*), the end-to-end distance of the linker, *L*, is calculated by 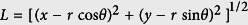 (Fig. 1b). For this length *L*, the pulling force imposing on the 5′-domain by the stretched linker, *f* (*L*), is calculated by Eq. (1), with its components in the *x* and *y* directions having forms 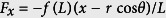 and 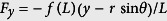, respectively. The finally active state of the 5′-domain is at (*x*, *y*, *θ*) = (−*r, d, π*), where *d* is the center-of-mass position of the 5′-domain in the *y* direction, which is equal to the end-to-end distance *L* of the linker at the finally active state (Fig. 1c). Here, we take *d* as a variable parameter. The interaction potential of the 5′-domain with the flap DNA can be written as


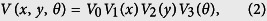


where *V*_0_ is the interaction strength of the 5′-domain with the flap DNA. *V*_1_(*x*), *V*_2_(*y*) and *V*_3_(*θ*) have the Morse-like forms


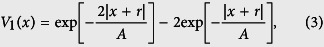



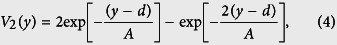



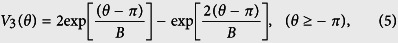


where *rB* = *A* = 0.5 nm that is consistent with the Debye length in the order of 1 nm in solution.

The motion and rotation of the 5′-domain in viscous solution can be described by Langevin equations


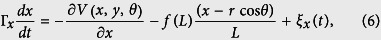



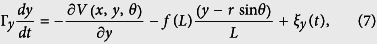



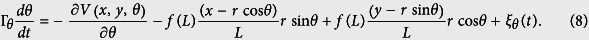


Here, the drag coefficients Γ_*x*_ = Γ_*y*_ = 6*πηr* = 6.59 × 10^−11^ kg ⋅ s^−1^ and Γ_*θ*_ = 8*πηr*^3^ = 1.0766 × 10^−9^ nm^2^ ⋅ kg ⋅ s^−1^, where to be consistent with the size of the enzyme (1TAQ.pdb) we take *r* = 3.5 nm, and the viscosity of the aqueous medium *η* = 0.01 g ⋅ cm^−1^ ⋅ s^−1^. The Langevin forces satisfy 〈*ξ*_*i*_(*t*)〉 = 0 (*i* = *x*, *y*, *θ*), 〈*ξ*_*i*_(*t*) *ξ*_*j*_(*t*')〉 = 0 (*i* ≠ *j*) and 〈*ξ*_*i*_(*t*) *ξ*_*i*_(*t*')〉 = 2*k*_*B*_*T* Γ_*i*_*δ*(*t−t*'). Without loss of generality^22^, we take the potential depth *V*_0_ = 18*k*_*B*_*T*. Here, we solve equations (6)–(8), 10, 11 numerically by using stochastic Runge-Kutta method, as done elsewhere^22^,^24^. To check if our simulations with this simple method are consistent with the all-atom MD simulations, we compare the results of short simulation time between the two methods (Fig. S2a), showing that the results with the two methods are consistent with each other.

Figure 2e shows a typical trajectory of the motion and rotation of the 5′-domain, where we take *d* = 4 nm. It is seen that the 5′-domain moves and rotates randomly until it binds stably to the flap at about 2.5 μs, implying that under this realization of simulations the transition time of the 5′-domain from the initial to final positions is about 2.5 μs. By averaging the transition times of 1000 realizations we obtain the mean transition time *T*_*m*_. It is noted that *T*_*m*_ is only sensitively dependent on the final state while is nearly independent of the initial state (Fig. S3).

In Fig. 2f we show *T*_*m*_ versus Δ*N* for different values of *d*. It is seen that for a fixed Δ*N*, as the value of *d* increases, the mean transition time *T*_*m*_ also increases. More interestingly, for a fixed *d*, there exists a critical value of Δ*N*, which is denoted by Δ*N*_*c*_. When Δ*N* is smaller than the critical value Δ*N*_*c*_, the transition time *T*_*m*_ increases significantly with the further decrease of Δ*N*. However, when Δ*N* ≥ Δ*N*_*c*_, the transition time is kept nearly unchanged with Δ*N*. For example, when *d* = 3.8 nm, Δ*N*_*c*_ = −1; when *d* is about 3.9 nm, Δ*N*_*c*_ = 0. This implies that if in the final state the linker is required to stretch to a length of about 3.9 nm, for the linker with the residue number equal to or larger than 16 (with Δ*N* = 0), the transition time *T*_*m*_ is kept to the minimum value; and if the residue number is smaller than 16 the transition time *T*_*m*_ increases significantly. It is noted that if the transition time is too long, the DNA substrate can be dissociated from PolI before the 5′-domain transits to the finally active mode. Thus, the 5′-domain should use the shortest time to transit to the active mode. Based on the above results, we see that for the finally stretched length of *L* = 3.9 nm, the number of residues in the linker should be *N *≥ 16, with *N* = 16 being the minimum number. Thus, for the wt linker having 16 (the minimum number) residues, it is expected that the linker should be stretched to a length of about 3.9 nm in the finally active mode. On the other hand, from the available structural data it is inferred that in the finally active mode the end-to-end distance of the linker is approximately 3.92 nm (Fig. 1c), which is in agreement with the value of about 3.9 nm inferred from our calculated results.

It should be mentioned here that for simplicity, in the above we have made calculations in two-dimensional (2D) space. Since in reality the 5′-domain moves in 3D space, to verify if our results in 2D space are applicable, we also make calculations in 3D space and compare with those in 2D space (see Section S1 and Fig. S2b in Supplementary Information). Our calculations show that although value of *T*_*m*_ in 3D space is evidently larger than the corresponding one in 2D space, which is consistent with previous theoretical analyses^25^,^26^, the features of *T*_*m*_ versus Δ*N* are similar for the two cases and the critical value Δ*N*_*c*_ in 3D space is the same as that in 2D space. This is understandable because the motion in the *z* direction is symmetric to the *xoy*-plane and thus has no effect on the features of *T*_*m*_ versus Δ*N* except that *T*_*m*_ is increased. Since in this work we only concern the feature of *T*_*m*_ versus Δ*N* and in particular the critical value Δ*N*_*c*_, our results on the transition of 5′-domain in 2D space is applicable. As in this work we need to do a million times of simulations to obtain all mean values of *T*_*m*_ (the timescale of each simulation being in the order of 1 μs to 1 ms), in the following we will make simulations in 2D space to save simulation time.

### Dynamics of transition of ZBD relative to RPD from inert to active modes in T7 primase

**Figure 3:**
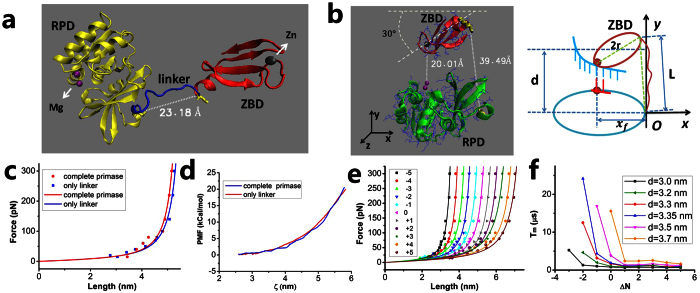
Structures, schematic diagrams and results for T7 primase. (**a**) Crystal structure of the enzyme (1NUI.pdb). (**b**) Left panel shows the most probable position and orientation of ZBD relative to RPD in the finally active mode. ZBD is in such position and orientation that the DNA-binding site of ZBD faces to the active site of RPD and the distance between them is approximately equal to 2 nm—the diameter of the DNA-RNA helix, because the active site of RPD should be coincident with the backbone of the base in the primer and the DNA-binding site of ZBD should be coincident with that of the opposite DNA base. In addition, in order to make the finally stretched length of the linker to be as short as possible, the rotation angle of the line that connects the DNA-binding site of ZBD and the position of the linker connecting to ZBD relative to the *x* axis to be as small as possible. Thus, we take the rotation angle to be *π*/6, giving *θ* = *π*/6 + *π* = 7*π*/6. Right panel is the schematic diagram of the structure shown in the left panel. (**c**) Force-extension relations of the linker simulated with complete enzyme and with only linker (dots). Lines are fit curves with WLC model. (**d**) PMF versus the end-to-end distance of the linker (*ζ*) simulated with complete enzyme and with only linker. (**e**) Force-extension relations of 11 linkers. (**f**) Mean transition time *T*_*m*_ versus Δ*N* for different values of *d*.

T7 primase is composed of RPD and ZBD domains that are connected by an unstructured polypeptide linker of 16 residues (Fig. 3a)^13^. To initiate the synthesis of primer, it is required that ZBD relative to RPD transits from an initially open conformation or inert state (Fig. 3a) to a finally closed conformation or active state (Fig. 3b) by stretching the linker. Thus, we should determine the elasticity of the linker before study the transition dynamics. As done in the case of PolI, we first determine if the presence of RPD and ZBD affects the elasticity of the linker. By using all-atom MD we make simulations of both the complete primase and only the linker. The results show that the force-extension curves for the two cases are nearly overlapped with each other (Fig. 3c). Furthermore, we calculate PMF during the pulling of the linker with the complete primase and with only the linker. The calculated results of PMF versus the end-to-end distance of the linker for the two cases are also nearly overlapped with each other (Fig. 3d). The above results thus indicate that the presence of RPD and ZBD has nearly no effect on the force-extension relation of the linker. Thus, as in the case of PolI, we can make simulations of only the linker to obtain the force-extension relation of the linker of T7 primase. As in the case of PolI, we calculate the force-extension curves for 11 linkers of different numbers of residues (Δ*N* = −5, …, +5), where residues are deleted from or added to the terminus (residue 70) of the wt linker and the added residues are chosen randomly. The calculated data of the force-extension relation can be fitted with WLC model (Fig. 3e).

Similar to the case of PolI, we approximate ZBD as a sphere with the radius of *r* and RPD as an ellipsoid. The relative motion between the two domains is studied by the motion of ZBD relative to the fixed RPD. The interaction potential of ZBD with DNA template is still described by equations (2)–(5), 14, 10, 11, and the motion and rotation of ZBD are still described by equations (6)–(8), 10, 11. To be consistent with the size of the enzyme (1NUI.pdb)^14^ we take *r* = 1.3 nm, giving drag coefficients Γ_*x*_ = Γ_*y*_ = 6*πηr* = 2.45 × 10^−11^ kg ⋅ s^−1^ and Γ_*θ*_ = 8*πηr*^3^ = 5.52 × 10^−11^ nm^2^ ⋅ kg ⋅ s^−1^. Based on the structures (Fig. 3a,b), the initial condition is (*x*, *y*, *θ*) = (*x*_0_, 0, 0) (*x*_0_ > *r*) and the final condition is (*x*, *y*, *θ*) = (−*x*_*f*_/2, *d*, 7*π*/6), where *θ* is defined as the rotation angle of the line connecting the center-of-mass position of ZBD and the DNA-binding site relative to the *y* axis, with *θ* = 0 in the initial state, *d* represents the distance between the center-of-mass position of ZBD and that of RPD in the finally active state, and *x*_*f*_ = 2 nm. Based on the structure (Fig. 3b), *d* is about 3.2 nm. Nevertheless, to see how the primase to use the minimum number of residues in the linker to achieve the most efficient transition, as done in the case of PolI we take *d* as a variable parameter but with fixed final rotation angle *θ* = 7*π*/6.

In Fig. 3f we show *T*_*m*_ versus Δ*N* for different values of *d*. The results are similar to Fig. 2f: for a fixed *d*, as Δ*N* becomes smaller than a critical value Δ*N*_*c*_ the transition time *T*_*m*_ increases rapidly with the further decrease of Δ*N*; and if Δ*N *≥ Δ*N*_*c*_ the transition time *T*_*m*_ has the minimum value independent of Δ*N*. In particular, we see that for *d* = 3.3 nm, Δ*N*_*c*_ = 0. This implies that the number of residues in the linker should be *N* ≥ 16 for the distance between the center-of-mass position of ZBD and that of RPD to be about *d* = 3.3 nm in the finally active state, with *N* = 16 being the minimum number. Thus, we expect that for the wt linker of 16 (the minimum number) residues, the linker is stretched to a length of about 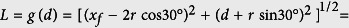
3.96 nm, which is in agreement with the value of about 3.95 nm deduced from the available structure (Fig. 3b).

### Dynamics of transition of LF domain from inactive to active modes in Dpo4

**Figure 4:**
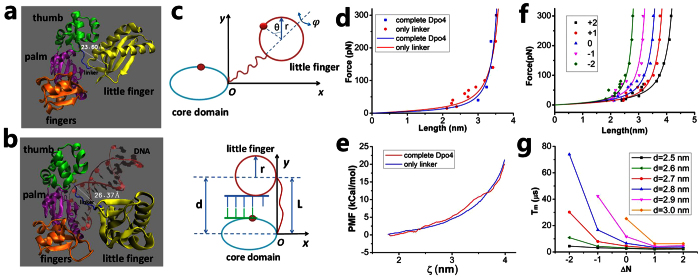
Structures, schematic diagrams and results for Dpo4. (**a**) Crystal structure of Dpo4 (2RDI.pdb). (**b**) Crystal structure of Dpo4 complexed with DNA in finally active state (3QZ7.pdb). (**c**) Schematic diagram of the system in intermediate and final states. (**d**) Force-extension relations of the linker simulated with complete Dpo4 and with only linker (dots). Lines are fit curves with WLC model. (**e**) PMF versus the end-to-end distance of the linker (*ζ*) simulated with complete Dpo4 and with only linker. (**f**) Force-extension relations of 5 linkers. (**g**) Mean transition time *T*_*m*_ versus Δ*N* for different values of *d*.

Dpo4 has a polymerase core consisting of a palm, fingers and thumb domain in addition to a LF domain. The polymerase core and the LF domain are connected by an unstructured polypeptide linker of 11 residues (Fig. 4a)^21^. Upon DNA binding, the LF domain relative to the polymerase core transits from the initially inactive to finally active states by stretching the liner (Fig. 4b). As for the case of PolI and T7 primase, in this section we study the effect of the linker on the transition of Dpo4 from the initially inactive to finally active states.

First, we determine the force-extension relation of the linker. As done above, we show that the presence of polymerase core and LF domains has nearly no effect on the elasticity of the linker (Fig. 4d,e). By simulating with only the linkers, we obtain the force-extension curves for 5 linkers of different residue numbers (Δ*N* = −2, …, +2), where residues are deleted from or added to the terminus (residue 244) of the wt linker and the added residues are chosen randomly. The calculated data of the force-extension relation can be fitted with WLC model (Fig. 4f).

As done above, we approximate LF domain as a sphere with the radius of *r* and the polymerase core as an ellipsoid (Fig. 4c). From the available structures^21^, it is noted that besides the motion and rotation of LF domain relative to the polymerase core, in the finally active state the LF domain also rotates along its azimuthal axis by about 131° relative to the initially inactive state. Thus, besides the motion in *Oxy* plane and rotation relative to the polymerase core (described by *θ*) we should also consider the azimuthal rotation, which is described by *ϕ* (Fig. 4c, upper panel). Similar to equation (2), the interaction potential of LF domain with DNA and the polymerase core can be written as *V*(*x*, *y*, *θ*, *ϕ*) = *V*_0_*V*_1_(*x*)*V*_2_(*y*)*V*_3_(*θ*)*V*_4_(*ϕ*), where *V*_1_(*x*), *V*_2_(*y*) and *V*_3_(*θ*) are still described by equations (3)–(5), 10, 11 and 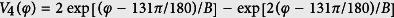 (0 ≤ *ϕ* ≤ 2*π*). The motion and rotation of LF domain are described by equations (6)–(8), 10, 11 supplemented by equation, Γ_*ϕ* _*dϕ*/*dt* = −∂*V*(*x*, *y*, *θ*, *ϕ*)/∂*ϕ* + *ξ*_*ϕ*_(*t*), with *ξ*_*ϕ*_ being the Langevin force. Here, considering that the linker is flexible, with a persistent length of only *L*_*p*_ = 0.3~0.4 nm (Fig. 4f), for approximation, we neglect the effect of the linker on the azimuthal rotation of LF domain.

To be consistent with the size of the enzyme (2RDI.pdb)^21^ we take *r* = 1.8 nm, giving drag coefficients Γ_*x*_ = Γ_*y*_ = 6*πηr* = 3.39 × 10^−11^ kg ⋅ s^−1^and Γ_*θ*_ = Γ_*ϕ*_ = 8*πηr*^3^ = 1.47 × 10^−10^ nm^2^ ⋅ kg ⋅ s^−1^. Based on the structures (Fig. 4a,b), the initial condition is (*x*, *y*, *θ*, *ϕ*) = (*x*_0_, 0, 0, 0) (*x*_0_ > *r*) and the final condition is (*x*, *y*, *θ*, *ϕ*) = (*−r*, *d*, *π*, 131*π/*180), where *d* is the center-of-mass position of LF domain in the *y* direction, which is equal to the end-to-end distance of the linker at the finally active state. As done above, we take *d* as a variable parameter.

In Fig. 4g we show *T*_*m*_ versus Δ*N* for different values of *d*, which are similar to Figs 2f and 3f. It is seen that for *d* = 2.7 nm, the critical value Δ*N*_*c*_ = 0. This implies that the number of residues in the linker should be *N *≥ 11 for the end-to-end distance of the linker to be about *d* = 2.7 nm in the finally active state, with *N* = 11 being the minimum number. Thus, we expect that for the wt linker of 11 (the minimum number) residues, the linker is stretched to a length of about *L* = *d* = 2.7 nm, which is consistent with the value of about 2.64 nm measured from the available structure (3QZ7.pdb)^21^ (Fig. 4b).

### The optimal number of residues in the linker

**Figure 5:**
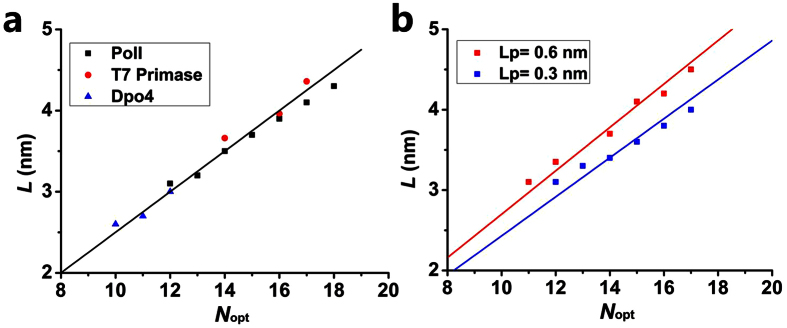
The relation of finally stretched length of the linker, *L*, with the optimal number of residues in the linker, *N*_*opt*_. (**a**) Dots are obtained from the results shown in Fig. 2f (data denoted by “PolI”), 3f (data denoted by “T7 primase”) and 4 g (data denoted by “Dpo4”). Line represents relation, *L* = *αN*_*opt*_, with *α* = 0.25 nm. (**b**) Dots are obtained from the results shown in Fig. S5 for the linkers with *L*_*p*_ = 0.3 nm (data denoted by “Lp = 0.3 nm”) and with *L*_*p*_ = 0.6 nm (data denoted by “Lp = 0.6 nm”). Lines are relation, *L* = *αN*_*opt*_, with *α* = 0.243 nm for data with *L*_*p*_ = 0.3 nm and *α* = 0.269 nm for data with *L*_*p*_ = 0.6 nm.

The linker of PolI is composed of 16 residues. In the active mode of the 5′-domain, the length of the linker is about *L* = 3.9 nm. Thus, on average, each residue has a length *α* = *L*/*N* = 0.244 nm along the direction that the linker is stretched, which is slightly smaller than the average size of about 0.33 nm for a residue. This implies that the linker is not completely stretched in the finally active position of the 5′-domain and thus, only a small internal force or a small increase in the internal free energy is induced, as noted from Fig. 2a,c. By contrast, if each residue is on average stretched to a length approaching 0.33 nm along the stretched direction, implying that the linker is nearly completely stretched, a very large internal force (>100 pN) or a very large increase of the internal free energy would be induced, making the final conformation be too unstable to perform the enzyme activity. T7 primase also has a linker of 16 residues. Although the size of ZBD of the primase is smaller than the 5′-domain of PolI, the finally stretched length of the linker is also about 3.96 nm, giving each residue to be stretched to a length of about 0.248 nm. The linker of Dpo4 consists of 11 residues and the finally stretched length of the linker is about 2.7 nm, giving each residue to be stretched to a length of about 0.245 nm. Thus, we note that although the linkers of the three enzymes have different numbers of residues, in the finally active mode each residue in the linker is stretched, on average, to a length of about 0.25 nm. More importantly, as our calculations show (Fig. S3), these results are independent of the initial length and conformation of the linkers. Thus, we conclude that at least for the systems studied in this work, the stretched length *L* of the linker in the finally active conformation and the optimal number (*N*_*opt*_) of the residues in the linker has the simple relation


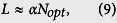


where *α* ≈ 0.25 nm. This can also be clearly seen from Fig. 5a, where we show the relation of *L* with *N*_*opt*_ by summarizing our results shown in Figs 2f,3f and 4g.

To generalize our conclusion, it is necessary to make further study of the effect of the sequence of residues in the linkers. For this purpose, we also calculate the force-extension relations of three linkers by mutating the wt linker of DNA polymerase I (Fig. S4). It is seen that the three force-extension curves of the mutant linkers are almost coincident with that of PolI and that of T7 primase, indicating that for the systems with the three mutant linkers the relation of *L* with *N*_*opt*_ has the same form as that for PolI and T7 primase. In addition, from the various previous studies^27^,^28^,^29^,^30^,^31^, it has been known that although different sequences may have different effects on their force-extension curves, the contour lengths *L*_*c*_ of different linkers have nearly the same value for a fixed number of residues and the persistence lengths *L*_*p*_ are almost always within a range of 0.3~0.6 nm^27^,^28^,^29^,^30^,^31^. Our results (Figs 2d,3e, 4f and S4) presented in this work give *L*_*p*_ = 0.3~0.4 nm, which is within the range of 0.3~0.6 nm. Furthermore, we check that even for the linkers with *L*_*p*_ = 0.3 nm and *L*_*p*_ = 0.6 nm, the length *L* and optimal number *N*_*opt*_ can still satisfy the relation given by equation (9), with *α* ≈ 0.243 nm and *α* ≈ 0.269 nm, respectively (Figs 5b and S5). Therefore, we conclude that for any enzyme whose two domains are connected by a flexible linker, if in the finally active conformation its linker is required to stretch to a length of *L*, the optimal number (*N*_*opt*_) of the residues in the linker can always be calculated by the simple relation of equation (9), with *α* = 0.24–0.27 nm being a constant insensitive to the system. If the number *N* is smaller than *N*_*opt*_, the transition from the inactive to active states would take too long time so that the substrate (e.g., DNA) bound to the enzyme is dissociated. If the number *N* is larger than *N*_*opt*_, the time for the enzyme to transit from the inactive to active states would be nearly the same as that with *N* = *N*_*opt*_. Thus, the redundant (*N*−*N*_*opt*_) residues would evolve to disappear.

## Conclusion

We study the dynamics of transition from the inactive to active states for PolI whose core and 5′-domains are connected by a linker of 16 residues, T7 primase whose ZBD and RPD domains are also connected by a linker of 16 residues, and Dpo4 whose polymerase and LF domains are connected by a linker of 11 residues. We show that the three enzymes share the same mechanism of transition from the inactive to active states. Moreover, we show that they use the minimum number of residues in the linker to achieve the most efficient transitions. More interestingly, we show that the finally stretched lengths (*L*) of the linker of the three enzymes in their active states and the optimal number (*N*_*opt*_) of the residues in the linker satisfy the relation *L* ≈ *αN*_*opt*_, with *α* being a constant of about 0.25 nm. Furthermore, our analysis show that for any enzyme whose two domains are connected by a flexible linker, the finally stretched length *L* and the optimal number *N*_*opt*_ of the residues in the linker satisfy the same relation, with *α* = 0.24–0.27 nm.

## Materials and Methods

The structures of PolI (1TAQ.pdb)^3^, T7 primase (1NUI.pdb)^14^ and Dpo4 (2RDI.pdb, 3QZ7.pdb)^21^ are taken from the RCSB protein data bank. The MD simulations are carried out by using GROMACS4.6 with AMBER99 force field. PMF is extracted from umbrella sampling simulations. The methods and parameter settings are the same as those in ref. 32. Further details are presented in SI text (Section S2: Extended methods).

## Additional Information

**How to cite this article**: Fu, Y.-B. *et al*. Optimal numbers of residues in linkers of DNA polymerase I, T7 primase and DNA polymerase IV. *Sci. Rep.* **6**, 29125; doi: 10.1038/srep29125 (2016).

## Supplementary Material

Supplementary Information
