# Supplementary material for: Optimal numbers of residues in linkers of DNA polymerase I, T7 primase and DNA polymerase IV
Source: Sci Rep. 2016 Jul 1;6:29125. doi: 10.1038/srep29125 (PMC4929570; doi:10.1038/srep29125)
Supplement: Supplementary Information [file srep29125-s1.doc]

**Supplementary Information**

**Optimal numbers of residues in linkers of DNA polymerase I, T7 primase and DNA polymerase IV**

Yi-Ben Fu, Zhan-Feng Wang, Peng-Ye Wang, Ping Xie

**SI text**

**S1. Dynamics of transition of 5**′**- domain in PolI in 3D space**

In the main text, the dynamics of transition of 5′-domain in PolI were studied in 2D space, where the interaction potential of the 5′-domain with the flap DNA was described by Eqs. (2) – (5), and the motion and rotation of the 5′-domain in viscous solution were described by Eqs. (6) – (8). Since the 5′-domain moves in 3D space and the kinetics of the diffusion are affected by the dimensionality [S1,S2], it is necessary to compare our simulation results in 2D space with those in 3D space, thus determining if our results in 2D space are applicable. Extending Eqs. (2) – (5), the interaction potential of the 5′-domain with the flap DNA in 3D space is written as

$V(x,y,\mathrm{z},\theta)=V_{0}V_{1}(x)V_{2}(y)V_{3}(\mathrm{z})V_{4}(\theta)$ , (S1)

$V_{1}(x)=\exp\left[-\frac{2\left|x+r\right|}{A}\right]-2\exp\left[-\frac{\left|x+r\right|}{A}\right]$, (S2)

$V_{2}(y)=2\exp\left[-\frac{(y-d)}{A}\right]-\exp\left[-\frac{2(y-d)}{A}\right]$, (S3)

$V_{3}(\mathrm{z})=-\exp\left[-\frac{2\left|z\right|}{A}\right]+2\exp\left[-\frac{\left|z\right|}{A}\right]$, (S4)

$V_{4}(\theta)=2\exp\left[\frac{(\theta-\pi)}{B}\right]-\exp\left[\frac{2(\theta-\pi)}{B}\right]$. $(\theta\geq-\pi)$ (S5)

Extending Eqs. (6) – (8), the motion and rotation of the 5′-domain in viscous solution in 3D space are described by

$\Gamma_{x}\frac{dx}{dt}=-\frac{\partial V\left(x,y,\mathrm{z},\theta\right)}{\partial x}-f\left(L\right)\frac{\left(x-r\cos\theta\right)}{L}+\xi_{x}\left(t\right)$, (S6)

$\Gamma_{y}\frac{dy}{dt}=-\frac{\partial V\left(x,y,\mathrm{z},\theta\right)}{\partial y}-f\left(L\right)\frac{\left(y-r\sin\theta\right)}{L}+\xi_{y}\left(t\right)$, (S7)

$\Gamma_{z}\frac{dz}{dt}=-\frac{\partial V\left(x,y,\mathrm{z},\theta\right)}{\partial z}-f\left(L\right)\frac{z}{L}+\xi_{z}\left(t\right)$, (S8)

$\Gamma_{\theta}\frac{d\theta}{dt}=-\frac{\partial V\left(x,y,\mathrm{z},\theta\right)}{\partial\theta}-f\left(L\right)\frac{\left(x-r\cos\theta\right)}{L}r\sin\theta+f\left(L\right)\frac{\left(y-r\sin\theta\right)}{L}r\cos\theta+\xi_{\theta}\left(t\right)$. (S9)

All parameters here are described in the main text. Additionally, the drag coefficients $\Gamma_{z}=\Gamma_{x}=\Gamma_{y}=6\pi\eta r=6.59\times10^{-11}\text{ kg}\cdot\text{s}^{-1}$, the end-to-end distance of the linker during the simulations is calculated by $L=[(x-r\cos\theta)^{2}+(y-r\sin\theta)^{2}+\mathrm{z}^{2}]^{\frac{1}{2}}$, the initial state of the 5′-domain is at (*x*,*y*,z,$\theta$) = (*x*_0_,0,0,0) (*x*_0_ > *r*), and the finally active state of the 5′-domain is at (*x*,*y*,z,$\theta$) = (*-r,d,*0*,π*).

In Fig. S2b we show the results of the mean transition time *T_m_* in 3D space versus $\Delta N$ for different values of *d* and the corresponding results in 2D space. It is seen that although value of *T_m_* in 3D space is evidently larger than the corresponding one in 2D space, which is consistent with the previous theoretical analyses [S1-S2], the features of *T_m_* versus $\Delta N$ are similar for the two cases and the critical value $\Delta N_{c}$ in 3D space is the same as that in 2D space. Since in this work we only concern the feature of *T_m_* versus $\Delta N$ and in particular the critical value $\Delta N_{c}$, our results on the transition of 5′-domain in 2D space is applicable.

**S2. Extended methods**

The MD simulations are carried out by using GROMACS4.6 [S3] with AMBER99 force field [S4]. To avoid the edge effect, the distance between the protein and the boundary of the box is at least 1 nm and much longer along the pulling force direction. We add solvent and necessary ions with favorable concentration. Counter-ions are also added to neutralize the system. All MD simulations are run at 300K and 1 bar. The time step is set as 2 fs, and the output data is updated every 5 steps. All chemical bonds are constrained using LINCS algorithm [S5]. The short range electrostatics interaction and the cutoff for van der Waals interaction is set as 1 nm. The long-range electrostatics interaction is calculated by using PME algorithm [S6]. Velocity-rescaling temperature coupling [S7] and Berendsen pressure coupling [S8] are used. The energy minimization is performed for 50000 steps by using the steepest descent method. Before the dynamic simulations, the systems are equilibrated successfully for 100 ps at 300 K and 1 bar pressure.

PMF is extracted from umbrella sampling simulations [S9]. To get the initial configurational windows for umbrella sampling simulations, some residues of the protein are fixed and some other residues are pulled under force that generates the extension of the linker. The single polypeptides are simulated in the similar ways except that the residues that are fixed and those on which the force are imposed are the terminal residues. The spring constant is $\text{1000 kJ}\cdot\text{mol}^{-1}\cdot\text{nm}^{-2}$ and pulling rate is $\text{0.001 nm}\cdot\text{ps}^{-1}$. From the configurational trajectory, snapshots are taken to generate the starting umbrella sampling windows. The spacing of the simulation windows is less than 0.2 nm and the simulation period lasts for 10 ns, so that the histograms of the configurations would sufficiently overlap with their neighbor windows. Results are analyzed using the weighted histogram analysis method [S10].

**References**

S1. Hardt, S.L. Rates of diffusion controlled reactions in one, two and three dimensions. *Biophys.* *Chem*. **10**, 239-243 (1979).

S2. Szabo, A., Schulten, K., Zan, S. First passage time approach to diffusion controlled reactions. *J. Chem. Phys.* **72**, 4350-4357 (1980).

S3. Hess, B., Kutzner, C., van der Spoel, D. & Lindahl, E. GROMACS 4: Algorithms for highly efficient, load-balanced, and scalable molecular simulation. *J. Chem. Theory Comput.* **4**, 435-447 (2008).

S4. Wang, J., Cieplak, P. & Kollman, P. A. How well does a restrained electrostatic potential (RESP) model perform in calculating conformational energies of organic and biological molecules? *J. Comp. Chem.* **21**, 1049-1074 (2000).

S5. Hess, B., Bekker, H., Berendsen, H. J. C. & Fraaije, J. G. E. M. LINCS: A linear constraint solver for molecular simulations. *J. Comput. Chem.* **18**, 1463-1472 (1997).

S6. Darden, T., York, D. & Pedersen, L. Particle mesh Ewald: An N log(N) method for Ewald sums in large systems. *J. Chem Phys* 98(12): 10089-10092 (1993).

S7. Bussi, G., Donadio, D. & Parrinello, M. Canonical sampling through velocity rescaling. *J. Chem. Phys.* **126**, 014101 (2007).

S8. Berendsen, H. J. C., Postma, J. P. M., Vangunsteren, W. F., Dinola, A. & Haak, J. R. Molecular dynamics with coupling to an external bath. *J. Chem. Phys.* **81**, 3684-3690 (1984).

S9. Torrie, G. M. & Valleau, J. P. Nonphysical sampling distributions in Monte Carlo free-energy estimation: Umbrella sampling. *J. Comput. Phys.* **23**, 187-199 (1977).

S10. Kumar, S., Bouzida, D., Swendsen, R. H., Kollman, P. A. & Rosenberg, J. M. The weighted histogram analysis method for free-energy calculations on biomolecules. I: The method. *J. Comput. Chem.* **13**, 1011-1021 (1992).

**SI figures**


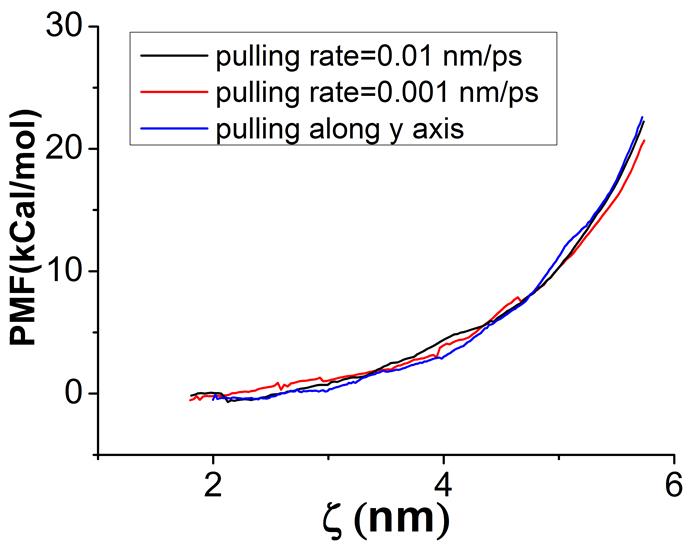


**Fig. S1. PMF of pulling the linker of PolI with different pulling rates and along different directions.** The black and red PMF curves are calculated by pulling the linker along the *z* axis, and the blue curve is calculated by pulling the linker along the *y* axis using the pulling rate of 0.001 nm/ps.


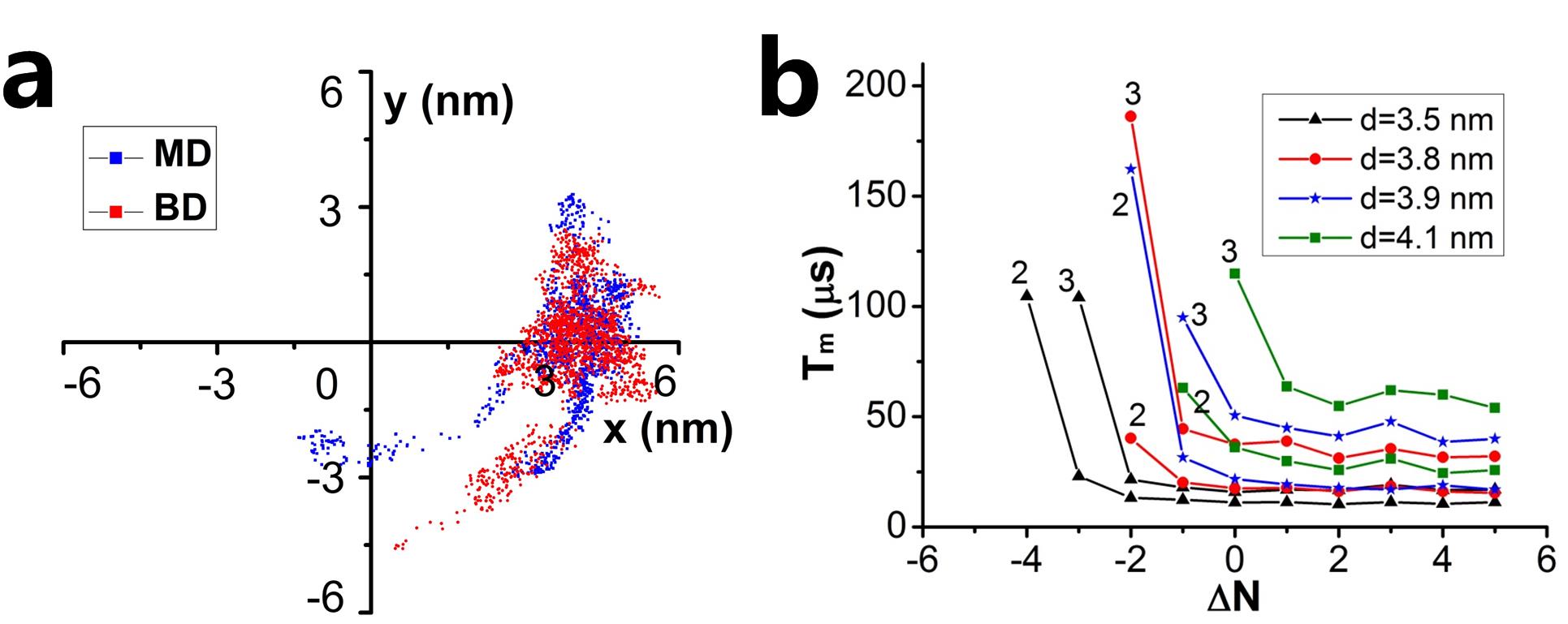


**Fig. S2. Validity of 2D model.** (a) Trajectories of 5′-nuclase domain of PolI are obtained from 7 realizations of simulation time of 20 ns, with each realization starting from the initial condition shown in Fig. 1a. Blue dots represent the data with all-atom MD simulations (denoted by MD), while red dots represent the data with Brownian dynamics simulation in 2D space, i.e., with numerical solution of equations (6) – (8) (denoted by BD). (b) Mean transition time *T_m_* versus $\Delta N$ in 3D space for different values of *d* and the corresponding results in 2D space. The numbers 2 and 3 in the figure represent 2D and 3D spaces, respectively.


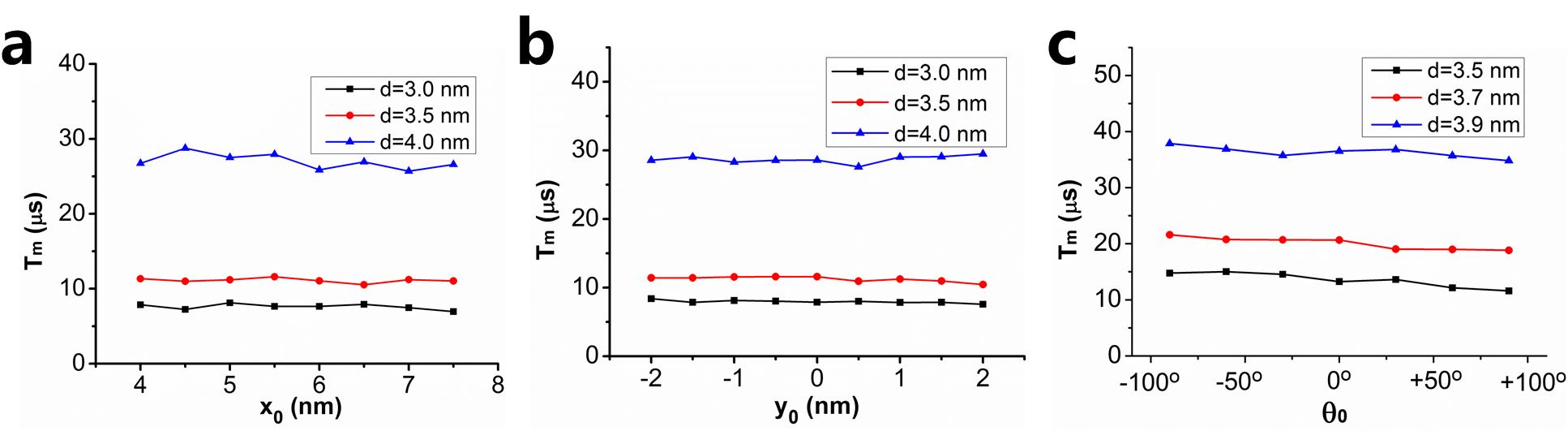


**Fig. S3. Variations of initial conditions on mean transition time *T_m_* with different values of *d* for PolI.** (a) *T_m_* versus *x*_0_ with fixed *y*_0_ = 0 and $\theta_{0}$ = 0. (b) *T_m_* versus *y*_0_ with fixed *x*_0_ = 5.64 nm and $\theta_{0}$ = 0. (c) *T_m_* versus $\theta_{0}$ with fixed *x*_0_ = 4 nm and *y*_0_ = 0.


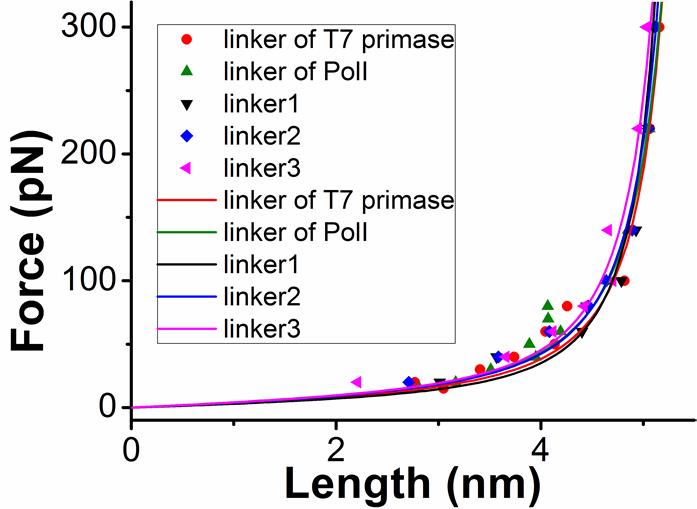


**Fig. S4. Force-extension relations of linkers of different sequences.** The sequence of the linker of PolI: SPKALEEAPWPPPEGA, the sequence of the linker of T7 primase: KRKPSGGKPGTYNVWN, the sequence of mutant linker1: SPKALEERPWPPPEGA, the sequence of mutant linker2: SPKRLEERPWPPPEGA, and the sequence of mutant linker3: SPKRLEERPWPRPEGA, where red boxes represent mutated residues.


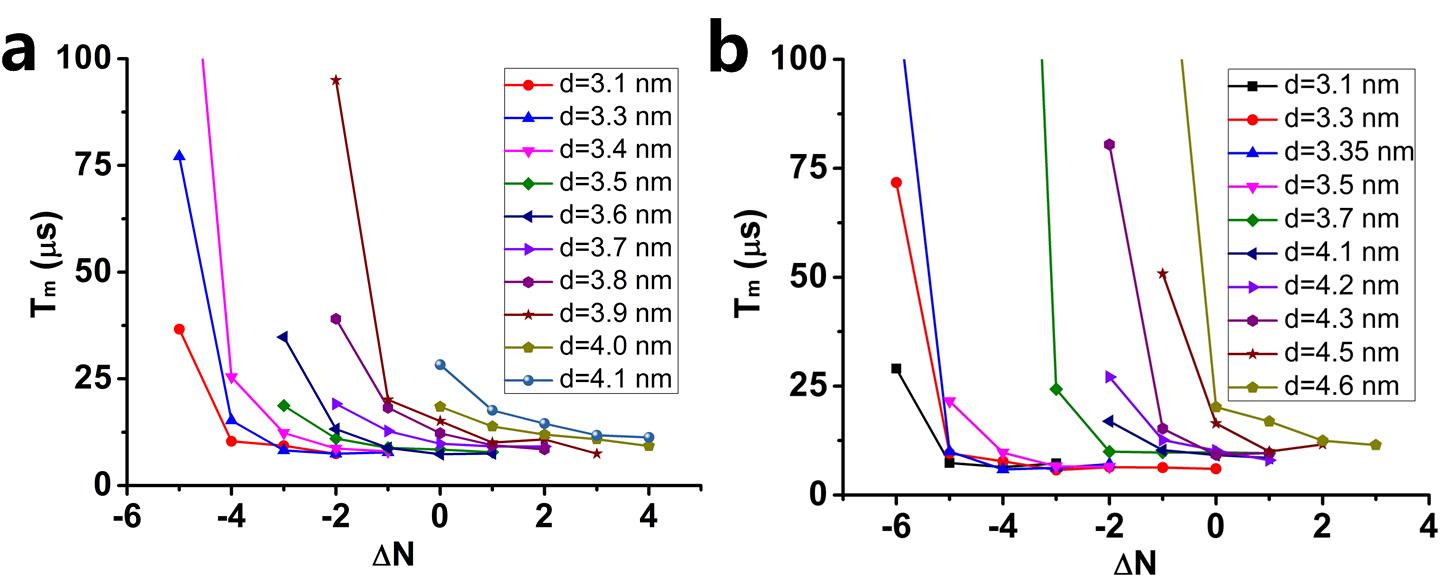


**Fig. S5. Results with linkers of *L_p_* = 0.3 nm and *L_p_* = 0.6 nm.** (a) Mean transition time *T_m_* versus $\Delta N$ for different values of *d* with *L_p_* = 0.3 nm. (b) Mean transition time *T_m_* versus $\Delta N$ for different values of *d* with *L_p_* = 0.6 nm.
